# Probing the Texture of the Calamitic Liquid Crystalline Dimer of 4-(4-Pentenyloxy)benzoic Acid

**DOI:** 10.3390/ma3020827

**Published:** 2010-01-29

**Authors:** Maher A. Qaddoura, Kevin D. Belfield

**Affiliations:** 1 Department of Chemistry, University of Central Florida, P.O. Box 162366, Orlando, FL 32816-2366, USA; 2 CREOL, The College of Optics and Photonics, University of Central Florida, P.O. Box 162366, Orlando, FL 32816-2366, USA

**Keywords:** liquid crystalline texture, nematic phase, smectic phase

## Abstract

The liquid crystalline dimer of 4-(4-pentenyloxy)benzoic acid, a member of the *n*-alkoxybenzoic acid homologous series, was synthesized using potassium carbonate supported on alumina as catalyst. The acid dimer complex exhibited three mesophases; identified as nematic, smectic X1 and smectic X2. Phase transition temperatures and the corresponding enthalpies were recorded using differential scanning calorimetry upon both heating and cooling. The mesophases were identified by detailed texture observations by variable temperature polarized light microscopy. The nematic phase was distinguished by a fluid Schlieren texture and defect points (four and two brushes) while the smectic phases were distinguished by rigid marble and mosaic textures, respectively.

## 1. Introduction

The objective of this work is to observe and investigate the texture of the liquid crystalline dimer of 4-(4-pentenyloxy)benzoic acid using polarized light microscopy in an attempt to understand the nature of the dramatic phase transitions during the heating and cooling cycles.

Although X-ray investigations have been successfully used to investigate the liquid crystalline phase transitions in particular the tilt angle, the determination of the phase has to be performed on macroscopically well oriented samples, which is time consuming and hard to accomplish. Both DSC and polarized light microscopy can be convenient and powerful tools to investigate these phases to adequate certainty.

Once the liquid crystalline material is heated, phase transitions occur based on the intrinsic properties of the compound that are characterized by the arrangement of the molecules, the conformation of the molecules, and intermolecular interactions. Typically, most liquid crystalline compounds go through different melting transitions called smectic and nematic phases. The difference between the two phases is primarily related to the arrangement of the molecules within the crystalline structure (packing order).

The scenario of transitions between crystalline-nematic-smectic phases occurs at defined temperatures and requires, as the first step, the breakdown of the crystalline order of the solid, causing oscillation, or rapid rotation about a given axis (usually the long axis of the molecules). Next, the long-range positional order is lost resulting in a smectic liquid crystal mesophase, which shows only orientational and short-range positional order within the diffused layers. The local packing order is then destroyed, except the orientational order on the long axes direction (known as the director of the phase), to give the nematic phase. Finally, all order is lost, forming the isotropic liquid [1,2]. Normally, one or more smectic phases might be revealed during the melting process; smecic A, smectic B, and smectic C are the most common smectic phases, differing only in their molecular packing order. However, some compounds undergo transition from crystalline to nematic without evidence of any smectic properties.

Calamitic (rod-like) liquid crystals based on *p*-alkoxybenzoic acid were investigated earlier for their liquid crystalline properties [3,4]; and have recently gained increased interest for the design of a variety of liquid crystalline compounds [5,6,7,8,9]. Alkenyloxybenzoate monomers are a functionalized version of alkoxybenzoates, and, due to the alkene functionality at the molecule’s terminus, has also gained special attention in designing functionalized liquid crystalline materials; such as, liquid crystalline elastomers [5,6,7,8,9]. Many physical properties of alkoxy and alkenoxy benzoic acid structures have been investigated, including their properties in binary systems [10], intermolecular hydrogen bonding formation [11,12,13], IR, Raman and thermodynamic investigations [14], the temperature dependence of their Raman spectra [15], the degree of order determination using IR dichroism [16], dielectric anisotropy [17] and their self assembly [18]. However, no single reference was found devoted to the in depth investigation of their texture under polarized light microscopy during both heating and cooling cycles. In the work reported herein, the thermal behavior and indepth texture observation of 4-(4-pentenyloxy) benzoic acid were investigated using both differential scanning calorimetry and optical polarized light microscopy, respectively.

## 2. Results and Discussion

### 2.1. Synthesis and thermal behavior of of 4-(4-pentenyloxy)benzoic acid *(**2**)*

**Scheme 1:**
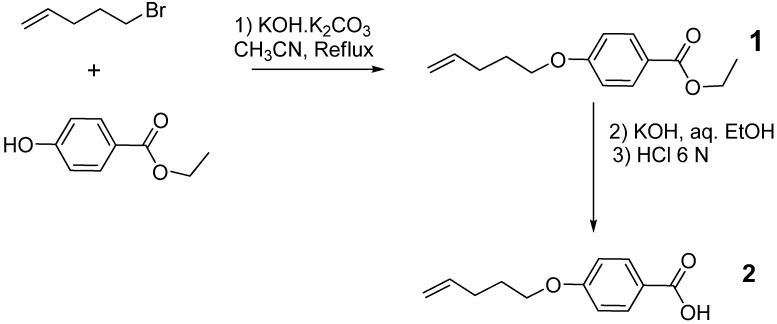
Monomer synthesis, 4-(4 -pentenyloxy)benzoic acid).

**Figure 1:**
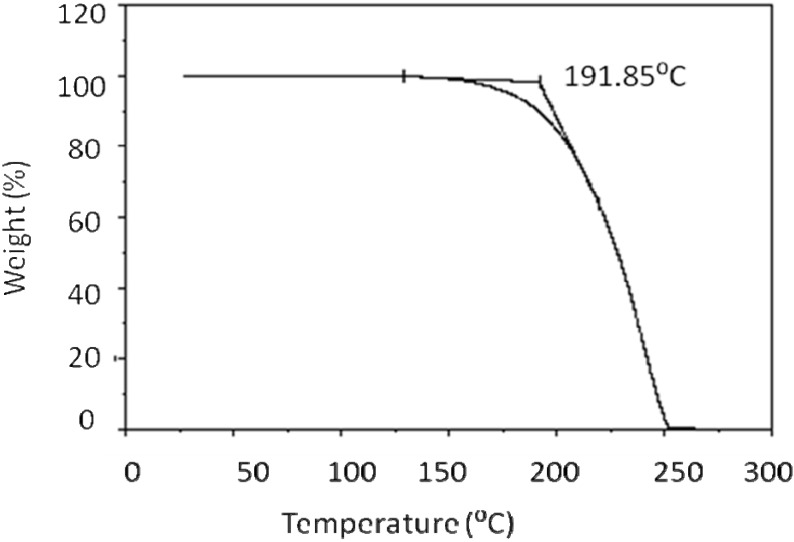
Thermogravemetric analysis of monomer **2**.

The synthesis of compound **2**; shown in Scheme 1, was carried out via a modified procedure. Ethyl 4-hydroxybenzoate was etherified with 5-bromo-1-pentene in the presence of a catalyst system of potassium carbonate supported on alumina followed by a hydrolysis using KOH then precipitation using HCl. This system gave a high yield of ester **1** in 4 h. This is significant since many researchers [19,20,21,22] have used different catalyst systems, such as K_2_CO_3_ in acetone or KOH in ethanol, producing lower yields and requiring longer reaction times (typically 12–48 h). ^1^H- and ^13^C-NMR spectra were consistent with the structure for **1**. The thermogravimetric analysis (Figure 1) indicated that the monomer began to decompose at about 192 ^○^C.

**Figure 2:**
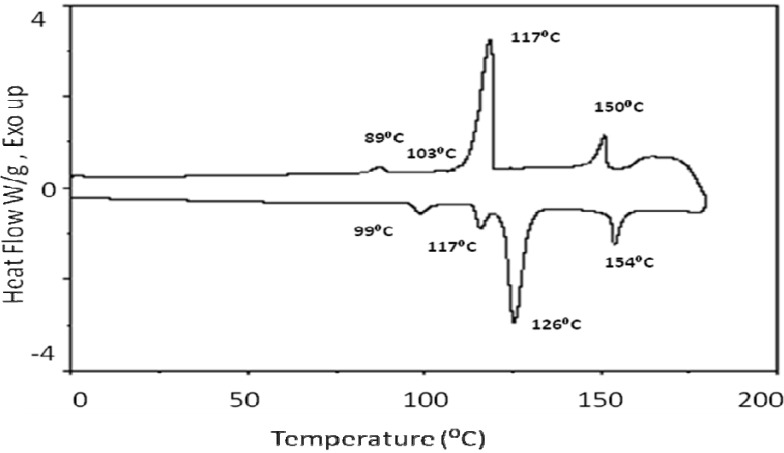
DSC thermograms for 4-(4-pentenyloxy) benzoic acid (**2**).

Figure 2 presents DSC thermogram of the acid compound **2**. It displayed several phases on both heating and cooling. Transition temperatures and enthalpy values are shown in Table 1. At 99 ^○^C, only 1.14 kJ mol^-1^ was released, indicating a small disruption of the packing order of the liquid crystal due to the translational and rotational action about the long access of the molecule. Thus, the crystalline-smectic transition required only small amount energy. At 117 ^○^C, only 1.4 kJ mol^-1^ was released due to what is ascribed to a smectic-smectic transition; while at 126 ^○^C, a significantly larger amount of energy was released 14.2 kJ mol^-1^, consistent with a major disruption in the molecular packing order; in particular, the positional order, while maintaining the orientational order. At 154 ^○^C, the molecules lose all types of order and become an isotropic liquid.

**Table 1:** Phase transition temperatures and enthalpy changes of compound **2**.

*Heating*		
**Phase Transition**	**Temperature ^○^C**	**Δ H kJmol^-1^**
Cr-SmX1	99	1.14
SmX1-SmX2	117	1.43
SmX2-N	126	14.2
N-I	154	2.9
*Cooling*		
**Phase Transition**	**Temperature ^○^C**	**Δ H kJmol^-1^**
I-N	150	-2.80
N-SmX1	117	-15.0
SmX1-SmX2	103	Very small
SmX2-Cr	89.0	-0.40

I: isotropic, N:nematic, SmX1: smectic X1, SmX2: smectic X2, Cr: crystalline.

**Figure 3:**
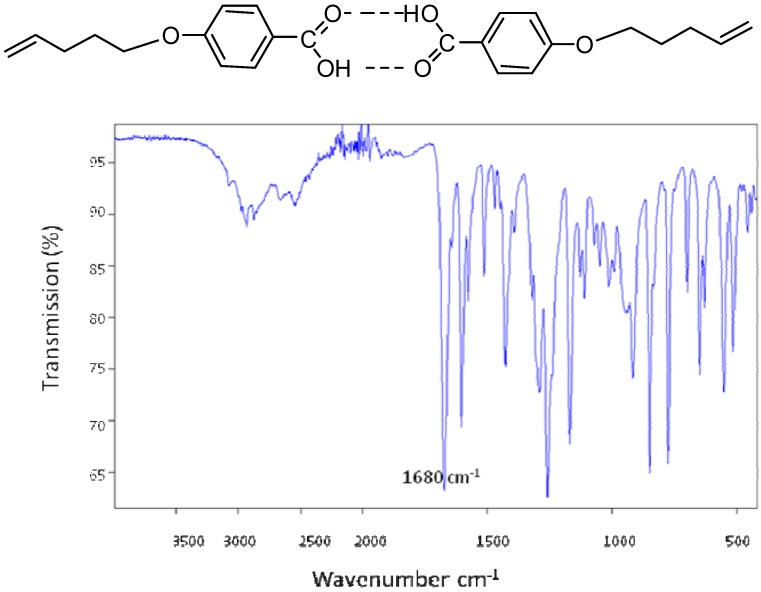
The proposed dimeric structure of 4-(4-pentenyloxy) benzoic acid; the spectrum indicates the presence of the carboxylic acid dimer; the carbonyl group in the proposed dimer structure has a characteristic band at 1680 cm^-1^.

According to the structure-phase relationships in liquid crystals, at least two ring structures are normally necessary to stabilize a calamitic liquid crystal phase. In this study, the monomer had a single benzene ring. The apparent contradiction of this principle by compound **2** is explained by dimer formation which has been reported for such type of structures [23,24,25,26,27,28]. The carboxylic acid dimer is formed via hydrogen bonding (Figure 3) [13]. This is supported by IR spectra analysis in which the carbonyl group in the dimer has a characteristic band at 1680 cm^-1^ [13,29], a significant shift from that of the free acid.

### 2.2. Texture study

**Figure 4:**
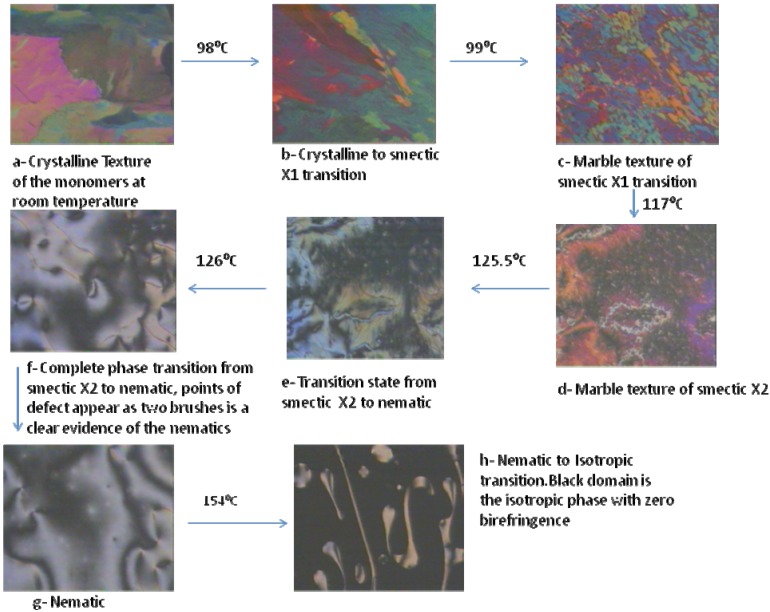
Liquid crystalline transitions for the compound **2** as observed under light polarized microscopy, crossed polarizer, magnification ×150.

Mesophase identification was determined by optical polarized light microscopic (PLM) observations; by increasing the temperature, sequences of phase transitions occurred that were consistent with the DSC investigations. Figure 4a shows the crystalline phase of compound **2**, while Figure 4b shows the beginning of a phase transition from crystalline to Smectic X1 (SmX1) upon heating up to 98 ºC. The complete transition to SmX1 took place at 99 ºC (Figure 4c). When the temperature was increased to 117 ºC, another transition was observed and identified as smectic X2 (SmX2), see Figure 4d. The transition from SmX2 to nematic starts to appear at 125.5 ºC (Figure 4e). At 126 ºC, (Figure 4f and Figure 4g), a complete transition to a nematic texture was observed, as can be seen from a Schlieren texture, points of extinction, and line defects. The Schlieren texture, observed between crossed polarizers, displayed dark brushes possessing an irregular curved shape, and corresponding to extinction positions of the nematic liquid. Accordingly, the director lies either parallel or normal to the polarizer or analyzer planes, respectively. At certain points, two dark brushes meet (Figure 4f and Figure 4g); at others there are four brushes coming together. These points indicate singularities, *i.e.,* disclination in structure [30,31].

Figure 4h illustrates a transition from the nematic to isotropic phase. This can be justified by understanding that nematic liquid crystals are optically uniaxial positive and strongly birefringent. Their refractive indices are very sensitive to temperature; the temperature coefficient being about a hundred times greater than that for a solid crystal. The ordinary index increases when the liquid crystal is heated, whereas the extraordinary index decreases. Thus, the birefringence starts to fall with a rise in temperature. At the nematic-isotropic liquid transition point there is a discontinuous change in the refractive indices which results in continous color change and the birefringence eventually drops abruptly to zero.

**Figure 5:**
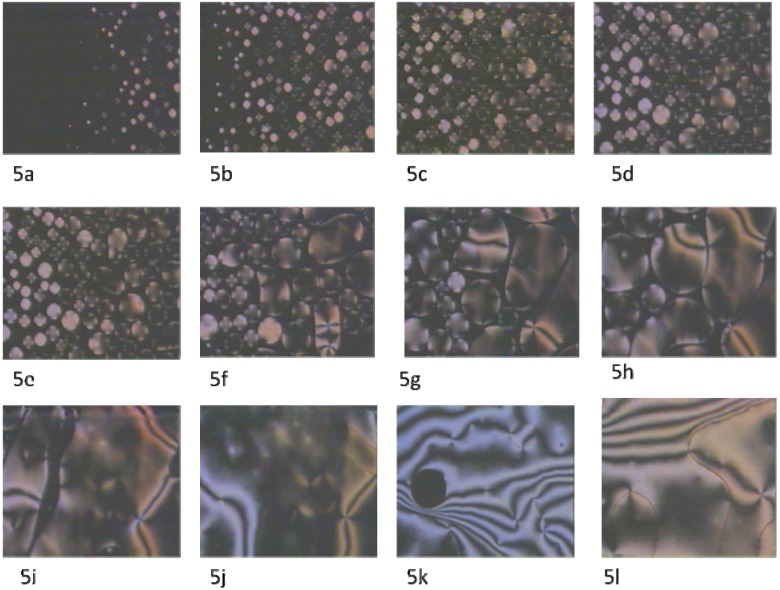
Phase transitions of the monomer upon cooling, cross polarizer, magnification ×150. (5a) Nematic droplets appearing from the isotropic phase with conoscopic crosses; (5b) nematic droplets continue growing at the clearing point with conoscopic crosses, 150 °C; (5c) nematic droplets are growing with increasing in size and show an optically positive uniaxial behavior, 150 °C; (5d) nematic droplets starts to coalesce with clear conoscopic crosses, 150 °C; (5e) coalescence of the crystalline germs,150 °C; (5f) coalescence of germs in the nematic phase and appearing of points singularities (four-fold brush), 150 °C; (5g) fourfolds brush defects start to appear, 150 °C; (5h) coalescence in progress, points of defect increase; 150 °C; (5i) nematic Schlieren texture grows from coalesced droplets, 150 °C; (5j) nematic Schlieren texture with four and two brushes, 150 °C; (5k) nematic Schlieren texture with air bubble, below 150 °C; (5l) nematic Schleiren texture with four and two brushes upon cooling, below 150 °C. In Figure 5k, a Schlieren texture with air bubble can be observed.

Upon cooling the isotropic phase of the monomer, nematic droplets start to appear from the isotropic phase. The nematic state at the clearing point starts to appear as round droplets. This can be clearly seen in Figure 5a and Figure 5b. When a system is quenched across a phase transition from the homogenous high temperature phase below the transition temperature T_c_, a nonequilibrium situation is created; crystalline germs of the low-temperature phase start to form nuclei and grow spontaneously. These observations can be applied to Figure 5b, Figure 5c, and Figure 5d. When these droplets become within interaction range of each other, they start to coalesce and form the nematic texture (Figure 5e, Figure 5f, Figure 5g, and Figure 5h).

**Figure 6:**
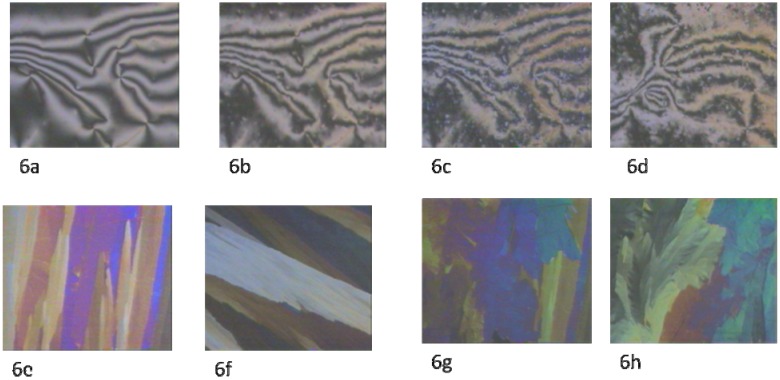
Transition from nematic to smectic to crystalline. (6a) Point singularities as evidence of nematic Schlieren texture, 150 °C; (6b) transition from nematic to smectic X2 upon cooling, 117 °C; (6c), (6d) smectic X2 phase, 117 °C; (6e), (5f) fast transition from smectic X2 to smectic X1. Mosaic texture of smectic X1, 103 °C; (6g) smectic X1 to crystalline phase transition upon cooling, 89 °C; (6h) crystalline phase; below 89 °C.

By further cooling to 117 ºC, a complete transition to SmX1 was observed at 103 ºC (see Figure 6b, Figure 6c and Figure 6d), a very fast transition occurred (Figure 6e), and a mosaic texture was observed. By further cooling, a crystalline phase emerged at 89 ºC (Figure 6f, Figure 6g, and Figure 6h).

**Figure 7:**
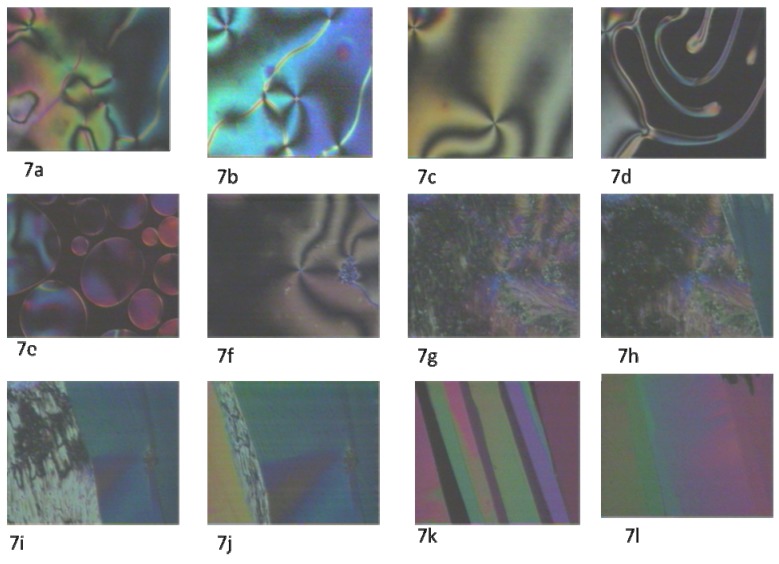
Phase transitions upon heating and cooling when external shear stress is applied under cross polarizer. (7a), (7b), and (7c), Nematic texture with: disclination lines (threads), points with extinction crosses, and wall defects, below 150 °C; (7d), nematic to isotropic transition, 150 °C; (7e) nematic droplets coalesce upon cooling, 150 °C; (7f) nematic Shlieren texture and points with extinction crosses, 145 °C; (7g) smectic X2 texture upon cooling, 117 °C; (7h) smectic X2 to smectic X1 transition upon cooling, 103 °C; (7i), (7j) smectic X2 to smectic X1 transition upon cooling, 103 °C; (7k), (7l) mosaic texture of smectic X1, 103 °C.

When applying external shear stress on the sample by rubbing on the surface of the coverslip during the nematic-isotropic transition, a flashy bright texture appeared. In Figure 7a, clear point singularities with extinction crosses, line defects, and wall defects (two-dimentional) were observed (see Figure 7a, Figure 7b, and Figure 7c). By bringing the temperature up to 154 ºC, a transition from nematic to isotropic occurred, where the birefringence dropped abruptly to zero (Figure 7d). Upon cooling, nematic droplets started to appear along with coalescence (Figure 7e). Four-brush defects can be clearly seen along with wall defects (Figure 7f). Figure 7g to Figure 7l illustrate dramatic transitions upon cooling from nematic to SmX2, SmX2 to SmX1, and SmX1.

**Figure 8:**
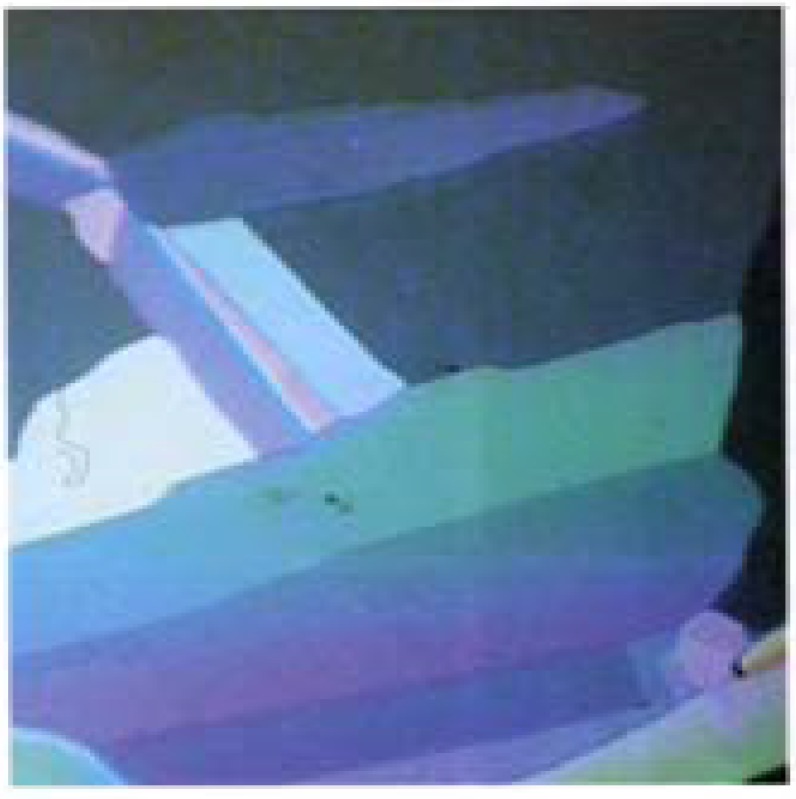
The mosaic texture is a common appearance of the smectic B phase as reported in the literature [40]. The mosaic textures are usually characterized by area of uniform optical appearance mediated by grain boundaries. The texture is very consistent with our observations in Figure 6e and Figure 7k.

In the structural units of the 4-*n*-alkoxybenzoic acids, the role of the dimer ring flexibility has still not been clarified [32]. It has been demonstrated, that for 2 < n < 6 (where n is the number of methylene units in the flexible alkyl tail), these compounds form nematic phases. These phases were found to exhibit pronounced optical properties of a chiral liquid crystal system, in our case this would be in the nematic phase. On further cooling, the chiral nematic phase undergoes a definite texture transition, becoming a normal nematic. In our case, we observed Sm X1. The explanation for this is that the dimer ring (interaction) can be destroyed at higher temperature and form a closed-open dimer. Formation of new hydrogen bonds is also possible by twisting around the –H^…^O axes of open dimers lying adjacent to each other and/or the simultaneous coupling between open dimers and monomers (oligomerization). This oligomerization process, which is a molecular organization stimulated by temperature variation and/or by surface action, could be the basis of achieving a spontaneous twist in a system of achiral molecules (supermolecular chirality). These phenomena have already been reported [33]. Such H-bonded supermolecular liquid crystals and their application in electro-optic devices recently gained some interest. Due to strong hydrogen bonding with the surface, it was suggested that a smectic B, can be generated [31]; this is consistent with what we have observed, i.e. the smectic X2 (see Figure 5e and Figure 6k) which is also consistent with the smectic B texture reported in the literature (see Figure 8). Moreover, in structures similar to alkoxybenzoic acid, similar work confirmed the possibility of induction of such smectic B phase [34], in addition to other phases like smectic E [34], smectic A [35,36,37,38], and crystal G [39].

## 3. Experimental Section

### 3.1. Materials

5-Bromo-1-pentene (97%) and potassium carbonate (99+%) were used as received from Lancaster. MN-Aluminum oxide, neutral (manufactured by Machery Nagel & Co) was used as received. Commercial methylene chloride was distilled from lithium aluminum hydride and stored over molecular sieves in a sealed container. THF was dried by distillation and stored over molecular sieves in a sealed container. HPLC grade acetonitrile was dried using molecular sieves and stored in a sealed container. All other reagents and solvents were commercially available.

### 3.2. Instrumentation

Thermogravimetric analysis (TGA) was performed using a Model 2050 thermogravimetric analyzer (*TA* Instruments) at a heating rate of 20 °C/min. The thermotropic behavior of all compounds was determined by a combination of differential scanning calorimetry (DSC) and polarized light microscopy. A Model 2920 differential scanning calorimeter (*TA* Instruments) was used to determine the thermal transitions, read as the maximum or minimum of the endothermic or exothermic peaks, respectively. All DSC heating and cooling rates were 10 °C/min. Thermal transitions were read from reproducible second or later heating scans and first or later cooling scans, respectively. Bruker optics vertex70 equipped with Helios FT-IR microsampler and Opus 5.5 software, was used for measuring the carboxylic acid dimer characteristic band. An Olympus BH-2 polarized optical microscope (magnification ×150), equipped with Mettler FP52 hot stage, was used to detect and image phase transitions. Thin samples were prepared by melting a minimum amount of compound between a clean glass slide and a cover slip. ^1^H- and ^13^C-NMR analyses were performed in CDCl_3_ (referenced to TMS at δ 0.0 ppm) on a Varian Gemini spectrometer (300 MHz for ^1^H and 75 MHz for ^13^C).

### 3.3. Synthesis

#### 3.3.1. Preparation of the K_2_CO_3_·Al_2_O_3_ Catalyst

Potassium carbonate (103 g, 0.74 mmol) was mixed with neutral aluminum oxide (150 g, 1.47 mmol) in distilled water (300 mL). This was stirred and heated to 50 °C for 1 h. The water was evaporated, and the resulting catalyst was activated overnight in an oven at 120 °C.

#### 3.3.2. Ethyl 4-(4-Pentenyloxy)benzoate (**1**)

A solution of 5-bromo-1-pentene (12.6 g, 85 mmol) was added dropwise to a refluxing solution of ethyl 4-hydroxybenzoate (15 g, 90 mmol) and (5 g) of potassium carbonate supported on alumina (the catalyst) . After 5 h of reflux, the reaction mixture was poured into water and extracted three times with methylene chloride (400 mL total). The organic layer was filtered to remove any traces of the catalyst. The solvent was removed on a rotary evaporator *in vacuo* to yield 19.2 g (97%) of ethyl 4-(4-pentenyloxy) benzoate as slightly yellowish oil. ^1^H-NMR (δ ppm TMS, CDCl_3_): 1.38 (t, CO_2_CH_2_C*H*_3_), 1.90 (m, C*H*_2_CH_2_O), 2.25 (q, C*H*_2_CH=), 4.02 (t, OC*H*_2_), 4.34 (q, CO_2_C*H*_2_), 5.05 (m, =C*H*_2_), 5.85 (m, =C*H*), 6.90 (d, 2 aromatic H ortho to OR), 7.98 (d, 2 aromatic H ortho to CO_2_R'). ^13^C-NMR (δ ppm TMS, CDCl_3_): δ 14.7, 28.6, 30.3, 60.8, 67.5, 114.1,115.5, 122.9, 131.6, 137.7, 162.9, 166.7.

#### 3.3.3. 4-(4-Pentenyloxy)benzoic Acid (**2**)

Ethyl 4-(4-pentenyloxy)benzoate (**1**, 19.2 g, 82 mmol) and potassium hydroxide (15 g, 0.27 mol) in ethanol (50 mL) and water (100 mL) were refluxed for 6 h. After being cooled to room temperature, the solution was acidified to pH 2 with concentrated HCl. The resulting precipitate was collected and recrystallized from ethanol, affording 15.2 g (90%) of 4-(4-pentenyloxy) benzoic acid (**1**) as white, shiny crystals. ^1^H-NMR (δ ppm TMS, CDCl_3_): 1.92 (m, C*H*_2_CH_2_O), 2.24 (q, C*H*_2_CH=), 4.04 (t, OC*H*_2_), 5.05 (m, =C*H*_2_), 5.86 (m, C*H*=), 6.93 (d, 2 aromatic H ortho to OR), 8.05 (d, 2 aromatic H ortho to CO_2_H). ^13^C-NMR (δ ppm TMS, CDCl_3_): δ 28.2, 30.0, 67.3, 114.0, 115.2, 121.3, 132.1, 137.3, 163.3, 171.8.

## 4. Conclusions

The monomer, 4-(4-pentenyloxy) benzoic acid (**2**), was synthesized using alumina-supported potassium carbonate catalyst. The thermal behavior of **2** was investigated using DSC, revealing the presence of several transitions; crystalline-smectic ×1, smectic ×1-smectic ×2, smectic ×2-nematic, and finally nematic-isotropic. The enthalpies of the transitions were calculated. The behavior was consistent with carboxylic acid dimer formation. The texture behavior of **2** was investigated in detail under crossed polarizers; the interaction between polarized light and distorted liquid crystals created birefringence, providing useful data about the phase structures. The nematic phase was characterized by two-fold and four-fold brushes (Schlieren texture), while the smectic phase exhibited a distinguished rigid mosaic texture.
